# 2-Amino­benzoic acid–4-[2-(pyridin-4-yl)eth­yl]pyridine (2/1)

**DOI:** 10.1107/S1600536813027128

**Published:** 2013-10-05

**Authors:** Hadi D. Arman, Edward R. T. Tiekink

**Affiliations:** a Department of Chemistry, The University of Texas at San Antonio, One UTSA Circle, San Antonio, Texas 78249-0698, USA; b Department of Chemistry, University of Malaya, 50603 Kuala Lumpur, Malaysia

## Abstract

The asymmetric unit of the title co-crystal, C_12_H_12_N_2_·2C_7_H_7_NO_2_, comprises a centrosymmetric 4-[2-(pyridin-4-yl)eth­yl]pyridine mol­ecule and a 2-amino­benzoic acid mol­ecule in a general position. The acid has a small twist between the carb­oxy­lic acid residue and the ring [dihedral angle = 7.13 (6)°] despite the presence of an intra­molecular N—H⋯O(carbon­yl) hydrogen bond. Three-mol­ecule aggregates are formed *via* O—H⋯N(pyrid­yl) hydrogen bonds, and these are connected into supra­molecular layers in the *bc* plane by N—H⋯O(carbon­yl) hydrogen bonds and π–π inter­actions between pyridine and benzene rings [inter-centroid distance = 3.6332 (9) Å]. Layers are connected along the *a* axis by weak π–π inter­actions between benzene rings [3.9577 (10) Å].

## Related literature

For co-crystals of 2-amino­benzoic acid with pyridyl derivatives, see: Arman, Kaulgud *et al.* (2012 ▶); Arman, Miller *et al.* (2012 ▶). For the isostructural 4,4′-bipyridyl analogue, see: Arman & Tiekink (2013 ▶).
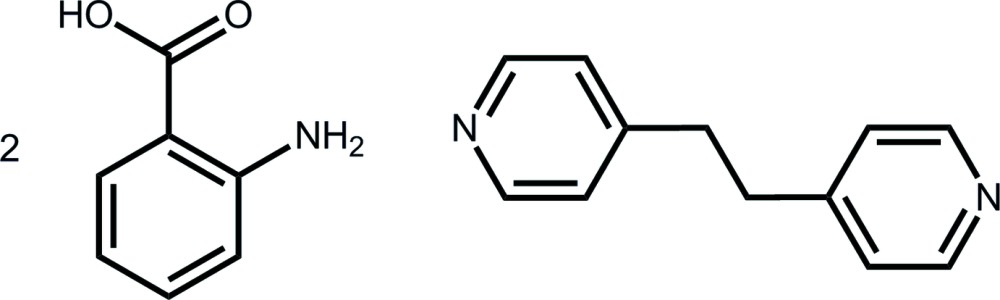


## Experimental

### 

#### Crystal data

C_12_H_12_N_2_·2C_7_H_7_NO_2_
*M* *_r_* = 458.51
Monoclinic, 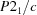

*a* = 11.305 (2) Å
*b* = 11.102 (2) Å
*c* = 8.8737 (16) Å
β = 94.565 (5)°
*V* = 1110.2 (4) Å^3^
*Z* = 2
Mo *K*α radiation
μ = 0.09 mm^−1^
*T* = 98 K
0.34 × 0.10 × 0.07 mm

#### Data collection

Rigaku AFC12/SATURN724 diffractometer
Absorption correction: multi-scan (*ABSCOR*; Higashi, 1995 ▶) *T*_min_ = 0.864, *T*_max_ = 1.000
8527 measured reflections
2545 independent reflections
2386 reflections with *I* > 2σ(*I*)
*R*_int_ = 0.039

#### Refinement

*R*[*F* ^2^ > 2σ(*F* ^2^)] = 0.045
*wR*(*F* ^2^) = 0.112
*S* = 1.08
2545 reflections
163 parameters
3 restraints
H atoms treated by a mixture of independent and constrained refinement
Δρ_max_ = 0.36 e Å^−3^
Δρ_min_ = −0.21 e Å^−3^

### 

Data collection: *CrystalClear* (Molecular Structure Corporation & Rigaku, 2005 ▶); cell refinement: *CrystalClear*; data reduction: *CrystalClear*; program(s) used to solve structure: *SHELXS97* (Sheldrick, 2008 ▶); program(s) used to refine structure: *SHELXL97* (Sheldrick, 2008 ▶); molecular graphics: *ORTEPII* (Johnson, 1976 ▶) and *DIAMOND* (Brandenburg, 2006 ▶); software used to prepare material for publication: *publCIF* (Westrip, 2010 ▶).

## Supplementary Material

Crystal structure: contains datablock(s) general, I. DOI: 10.1107/S1600536813027128/xu5744sup1.cif

Structure factors: contains datablock(s) I. DOI: 10.1107/S1600536813027128/xu5744Isup2.hkl

Supplementary material file. DOI: 10.1107/S1600536813027128/xu5744Isup3.cml

Additional supplementary materials:  crystallographic information; 3D view; checkCIF report

## Figures and Tables

**Table 1:** Hydrogen-bond geometry (Å, °)

*D*—H⋯*A*	*D*—H	H⋯*A*	*D*⋯*A*	*D*—H⋯*A*
N1—H1n⋯O2	0.86 (1)	2.03 (1)	2.6961 (15)	134 (2)
O1—H1o⋯N2	0.86 (1)	1.78 (1)	2.6290 (14)	172 (2)
N1—H2n⋯O2^i^	0.85 (1)	2.19 (1)	3.0106 (15)	163 (1)

Symmetry code: (i) 
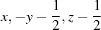
.

**Fig. 1.**
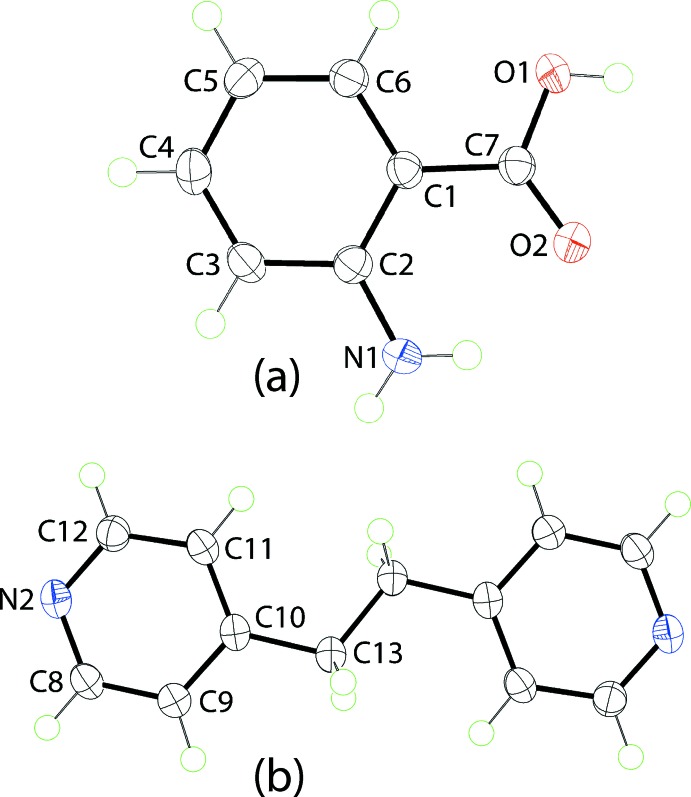
Molecular structures of the components of (I), showing atom-labelling scheme and displacement ellipsoids at the 70% probability level: (a) 2-aminobenzoic acid and (b) 4,4'-bipyridylethane (unlabelled atoms are related by the symmetry operation: 1 - x, -y, 2 - z).

**Fig. 2.**
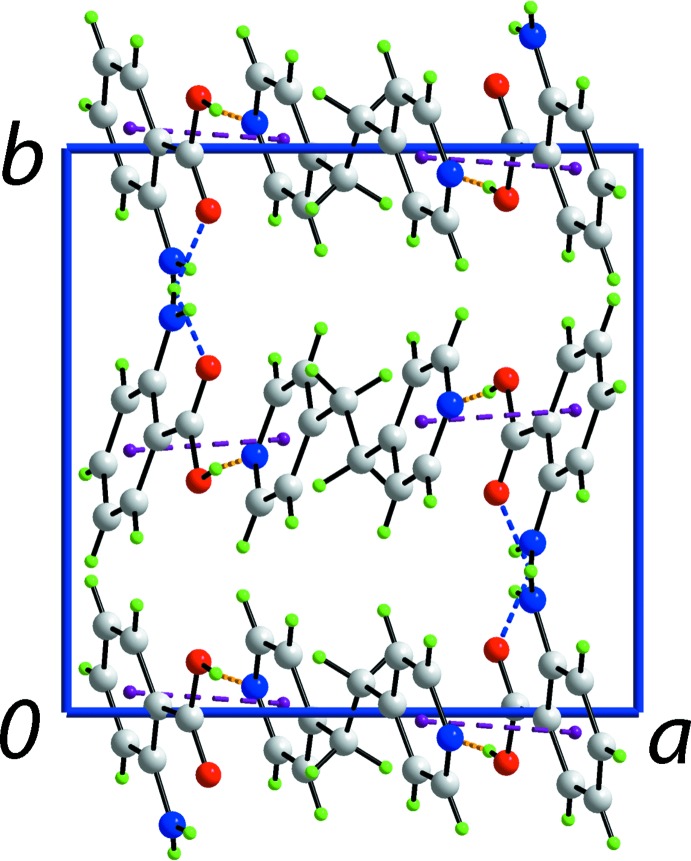
Side-on view of the supramolecular layer in the bc plane in (I). The three-molecule aggregates are sustained by O—H···N hydrogen bonds shown as orange dashed lines. These are connected into layers by N—H···O and π—π interactions, shown as blue and purple dashed lines, respectively.

**Fig. 3.**
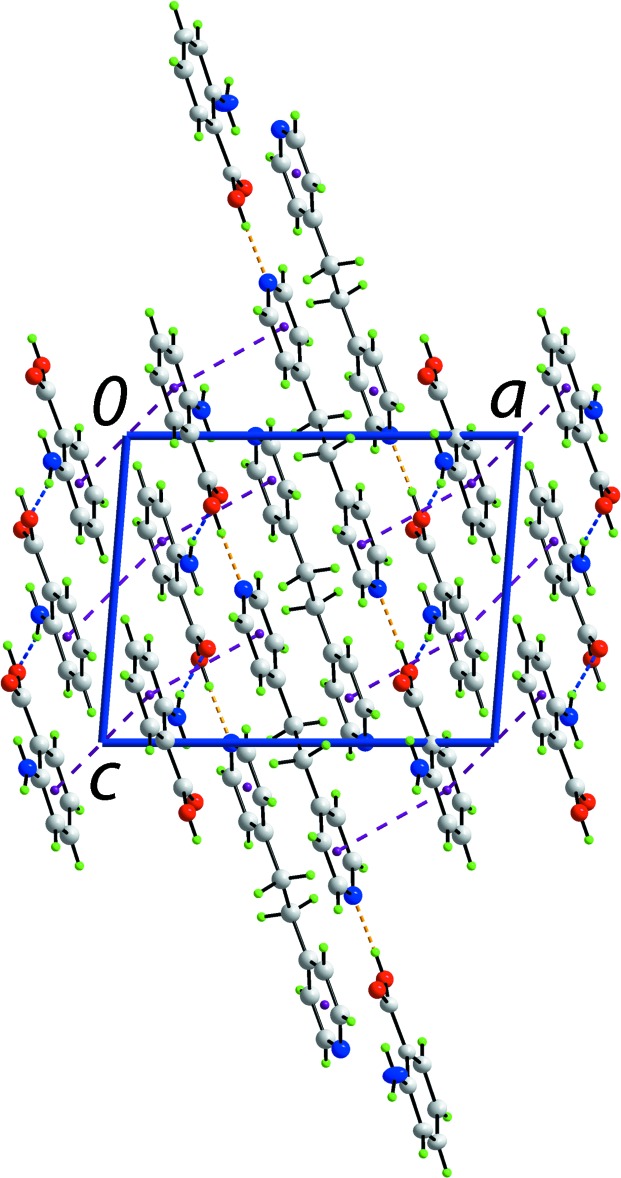
Unit-cell contents of (I) viewed in projection down the b axis. The O—H···N, N—H···O and π—π interactions are shown as orange, blue and purple dashed lines, respectively.

## supplementary crystallographic information

### 1. Comment

During continuing structural studies of co-crystals involving 2-aminobenzoic acid (anthranilic acid) and variously substituted pyridyl derivatives (Arman, Kaulgud *et al.*, 2012; Arman, Miller *et al.*, 2012), the title co-crystal, (I), was characterized.

The asymmetric unit of (I), Fig. 1, comprises a molecule of 2-aminobenzoic acid in a general position and half a molecule of 4,4'-bipyridylethane, being disposed about a centre of inversion. Despite the presence of an intramolecular N1—H···O2 hydrogen bond, Table 1, the carboxylic acid residue is slightly twisted out of the plane of the benzene ring to which it is connected, forming a dihedral angle of 7.13 (6)°. The 4,4'-bipyridylethane molecule is also almost planar with the r.m.s. deviation of the 14 non-hydrogen atoms being 0.066 Å. The structure of (I) is isostructural with the 4,4'-bipyridyl derivative (Arman & Tiekink, 2013).

The components of the co-crystal are connected into a three-molecule aggregate *via* O1—H···N2 hydrogen bonds, Table 1. These are connected into supramolecular layers in the *bc* plane by N1—H···O2 hydrogen bonds, Fig. 2. Additional stability to the layers is afforded by π—π interactions between the pyridyl and benzene rings [inter-centroid distance = 3.6332 (9) Å, angle of inclination = 1.71 (6)° for symmetry operation *x*, *y*, 1 + *z*], Fig. 2. Weaker π—π interactions between centrosymmetrically related benzene rings [3.9577 (10) Å for symmetry operation: -*x*, -*y*, -*z*] provide the links between the layers, Fig. 3.

### 2. Experimental

Crystals of (I) were obtained by the co-crystallization of 4,4'-bipyridylethane (Sigma Aldrich, 0.11 mmol) and anthranilic acid (Sigma-Aldrich, 0.22 mmol) in acetone solution. Crystals were obtained by slow evaporation. Melting point: 381–385 K. IR spectra(cm^-1^): 750(sh)(*m*), 830(*s*)(sh), 1016(w), 1063(w), 1153(*m*), 1238(*m*), 1295(*s*), 1413(*m*), 1607(*m*), 1661(*m*), 2922(br), 3342(*m*), 3449(*m*).

### 3. Refinement

C-bound H-atoms were placed in calculated positions (C—H = 0.95-0.99 Å) and were included in the refinement in the riding model approximation with *U*_iso_(H) set to 1.2*U*_eq_(C). The O-bound and N-bound H-atoms were located in a difference Fourier map and were refined with a distance restraints of O—H = 0.84±0.01 Å and N—H = 0.88±0.01 Å, and with *U*_iso_(H) = 1.2*U*_eq_(N) and 1.5*U*_eq_(O).

### Crystal data

**Table:**

C_12_H_12_N_2_·2C_7_H_7_NO_2_	*F*(000) = 484
*M**_r_* = 458.51	*D*_x_ = 1.372 Mg m^−^^3^
Monoclinic, *P*2_1_/*c*	Mo *K*α radiation, λ = 0.71069 Å
Hall symbol: -P 2ybc	Cell parameters from 3826 reflections
*a* = 11.305 (2) Å	θ = 2.6–40.2°
*b* = 11.102 (2) Å	µ = 0.09 mm^−^^1^
*c* = 8.8737 (16) Å	*T* = 98 K
β = 94.565 (5)°	Needle, gold
*V* = 1110.2 (4) Å^3^	0.34 × 0.10 × 0.07 mm
*Z* = 2	

### Data collection

**Table:**

Rigaku AFC12K/SATURN724 diffractometer	2545 independent reflections
Radiation source: fine-focus sealed tube	2386 reflections with *I* > 2σ(*I*)
Graphite monochromator	*R*_int_ = 0.039
ω scans	θ_max_ = 27.5°, θ_min_ = 2.6°
Absorption correction: multi-scan (*ABSCOR*; Higashi, 1995)	*h* = −14→14
*T*_min_ = 0.864, *T*_max_ = 1.000	*k* = −14→14
8527 measured reflections	*l* = −9→11

### Refinement

**Table:**

Refinement on *F*^2^	Primary atom site location: structure-invariant direct methods
Least-squares matrix: full	Secondary atom site location: difference Fourier map
*R*[*F*^2^ > 2σ(*F*^2^)] = 0.045	Hydrogen site location: inferred from neighbouring sites
*wR*(*F*^2^) = 0.112	H atoms treated by a mixture of independent and constrained refinement
*S* = 1.08	*w* = 1/[σ^2^(*F*_o_^2^) + (0.0469*P*)^2^ + 0.5018*P*] where *P* = (*F*_o_^2^ + 2*F*_c_^2^)/3
2545 reflections	(Δ/σ)_max_ < 0.001
163 parameters	Δρ_max_ = 0.36 e Å^−^^3^
3 restraints	Δρ_min_ = −0.21 e Å^−^^3^

### Special details

**Table:**

Geometry. All s.u.'s (except the s.u. in the dihedral angle between two l.s. planes) are estimated using the full covariance matrix. The cell s.u.'s are taken into account individually in the estimation of s.u.'s in distances, angles and torsion angles; correlations between s.u.'s in cell parameters are only used when they are defined by crystal symmetry. An approximate (isotropic) treatment of cell s.u.'s is used for estimating s.u.'s involving l.s. planes.
Refinement. Refinement of *F*^2^ against ALL reflections. The weighted *R*-factor *wR* and goodness of fit *S* are based on *F*^2^, conventional *R*-factors *R* are based on *F*, with *F* set to zero for negative *F*^2^. The threshold expression of *F*^2^ > 2σ(*F*^2^) is used only for calculating *R*-factors(gt) *etc*. and is not relevant to the choice of reflections for refinement. *R*-factors based on *F*^2^ are statistically about twice as large as those based on *F*, and *R*- factors based on ALL data will be even larger.

### Fractional atomic coordinates and isotropic or equivalent isotropic displacement parameters (Å^2^)

**Table:**

	*x*	*y*	*z*	*U*_iso_*/*U*_eq_	
O1	0.23122 (9)	0.08940 (8)	0.22847 (10)	0.0230 (2)	
H1O	0.2610 (14)	0.0684 (16)	0.3166 (13)	0.035*	
O2	0.24830 (8)	−0.11075 (8)	0.19402 (10)	0.0221 (2)	
N1	0.18511 (11)	−0.19962 (10)	−0.08352 (12)	0.0232 (3)	
H1N	0.2156 (13)	−0.2139 (15)	0.0061 (12)	0.028*	
H2N	0.1885 (14)	−0.2498 (12)	−0.1548 (14)	0.028*	
C1	0.15987 (10)	0.01116 (10)	−0.00795 (13)	0.0161 (2)	
C2	0.15020 (10)	−0.08349 (11)	−0.11662 (13)	0.0172 (2)	
C3	0.10295 (11)	−0.05498 (11)	−0.26429 (13)	0.0193 (3)	
H3	0.0973	−0.1160	−0.3396	0.023*	
C4	0.06456 (11)	0.06078 (12)	−0.30126 (13)	0.0208 (3)	
H4	0.0332	0.0776	−0.4016	0.025*	
C5	0.07110 (11)	0.15347 (11)	−0.19362 (14)	0.0204 (3)	
H5	0.0436	0.2322	−0.2196	0.024*	
C6	0.11832 (10)	0.12773 (11)	−0.04894 (13)	0.0181 (3)	
H6	0.1230	0.1899	0.0249	0.022*	
C7	0.21531 (10)	−0.01002 (11)	0.14633 (13)	0.0172 (2)	
N2	0.32920 (9)	0.04519 (10)	0.50153 (11)	0.0194 (2)	
C8	0.36977 (11)	−0.06728 (11)	0.53384 (13)	0.0199 (3)	
H8	0.3642	−0.1262	0.4559	0.024*	
C9	0.41896 (11)	−0.10008 (11)	0.67537 (13)	0.0195 (3)	
H9	0.4450	−0.1805	0.6939	0.023*	
C10	0.43024 (10)	−0.01417 (11)	0.79159 (13)	0.0175 (3)	
C11	0.38823 (11)	0.10211 (11)	0.75721 (13)	0.0189 (3)	
H11	0.3940	0.1633	0.8323	0.023*	
C12	0.33799 (11)	0.12738 (11)	0.61248 (13)	0.0197 (3)	
H12	0.3087	0.2063	0.5914	0.024*	
C13	0.48476 (11)	−0.05171 (11)	0.94524 (13)	0.0192 (3)	
H13A	0.5581	−0.0978	0.9316	0.023*	
H13B	0.4289	−0.1066	0.9916	0.023*	

### Atomic displacement parameters (Å^2^)

**Table:**

	*U*^11^	*U*^22^	*U*^33^	*U*^12^	*U*^13^	*U*^23^
O1	0.0346 (5)	0.0189 (5)	0.0145 (4)	0.0008 (4)	−0.0047 (4)	−0.0020 (3)
O2	0.0306 (5)	0.0181 (4)	0.0170 (4)	0.0008 (3)	−0.0013 (3)	0.0020 (3)
N1	0.0359 (6)	0.0157 (5)	0.0175 (5)	0.0027 (4)	−0.0014 (4)	−0.0031 (4)
C1	0.0178 (5)	0.0164 (6)	0.0141 (5)	−0.0013 (4)	0.0019 (4)	−0.0001 (4)
C2	0.0176 (5)	0.0167 (6)	0.0174 (5)	−0.0014 (4)	0.0023 (4)	0.0005 (4)
C3	0.0227 (6)	0.0200 (6)	0.0151 (5)	−0.0039 (5)	0.0009 (4)	−0.0026 (4)
C4	0.0219 (6)	0.0244 (6)	0.0156 (5)	−0.0023 (5)	−0.0015 (4)	0.0027 (4)
C5	0.0222 (6)	0.0176 (6)	0.0211 (6)	0.0022 (5)	0.0002 (4)	0.0031 (5)
C6	0.0205 (5)	0.0168 (6)	0.0171 (5)	−0.0005 (4)	0.0022 (4)	−0.0005 (4)
C7	0.0194 (5)	0.0176 (6)	0.0148 (5)	−0.0006 (4)	0.0027 (4)	0.0005 (4)
N2	0.0201 (5)	0.0232 (5)	0.0146 (5)	−0.0025 (4)	−0.0002 (4)	−0.0002 (4)
C8	0.0223 (6)	0.0206 (6)	0.0166 (5)	−0.0023 (5)	0.0009 (4)	−0.0032 (4)
C9	0.0208 (6)	0.0191 (6)	0.0184 (6)	0.0005 (4)	−0.0002 (4)	−0.0012 (4)
C10	0.0170 (5)	0.0209 (6)	0.0144 (5)	−0.0017 (4)	0.0003 (4)	0.0000 (4)
C11	0.0201 (6)	0.0198 (6)	0.0166 (5)	−0.0025 (4)	0.0002 (4)	−0.0028 (4)
C12	0.0212 (6)	0.0194 (6)	0.0184 (6)	−0.0013 (4)	0.0002 (4)	0.0009 (4)
C13	0.0213 (6)	0.0201 (6)	0.0156 (5)	0.0001 (5)	−0.0017 (4)	0.0002 (5)

### Geometric parameters (Å, º)

**Table:**

O1—C7	1.3271 (14)	C6—H6	0.9500
O1—H1O	0.859 (9)	N2—C12	1.3402 (16)
O2—C7	1.2425 (15)	N2—C8	1.3530 (16)
N1—C2	1.3732 (16)	C8—C9	1.3813 (17)
N1—H1N	0.856 (9)	C8—H8	0.9500
N1—H2N	0.845 (9)	C9—C10	1.4031 (16)
C1—C6	1.4144 (16)	C9—H9	0.9500
C1—C2	1.4246 (16)	C10—C11	1.4007 (17)
C1—C7	1.4783 (16)	C10—C13	1.5096 (16)
C2—C3	1.4111 (16)	C11—C12	1.3910 (16)
C3—C4	1.3875 (18)	C11—H11	0.9500
C3—H3	0.9500	C12—H12	0.9500
C4—C5	1.4020 (17)	C13—C13^i^	1.526 (2)
C4—H4	0.9500	C13—H13A	0.9900
C5—C6	1.3805 (16)	C13—H13B	0.9900
C5—H5	0.9500		
			
C7—O1—H1O	107.5 (12)	O1—C7—C1	113.90 (10)
C2—N1—H1N	117.4 (11)	C12—N2—C8	117.95 (10)
C2—N1—H2N	119.2 (11)	N2—C8—C9	122.76 (11)
H1N—N1—H2N	122.3 (16)	N2—C8—H8	118.6
C6—C1—C2	119.66 (11)	C9—C8—H8	118.6
C6—C1—C7	119.43 (10)	C8—C9—C10	119.70 (11)
C2—C1—C7	120.90 (11)	C8—C9—H9	120.1
N1—C2—C3	119.37 (11)	C10—C9—H9	120.1
N1—C2—C1	122.85 (11)	C11—C10—C9	117.20 (11)
C3—C2—C1	117.78 (11)	C11—C10—C13	123.93 (11)
C4—C3—C2	121.03 (11)	C9—C10—C13	118.87 (11)
C4—C3—H3	119.5	C12—C11—C10	119.56 (11)
C2—C3—H3	119.5	C12—C11—H11	120.2
C3—C4—C5	121.35 (11)	C10—C11—H11	120.2
C3—C4—H4	119.3	N2—C12—C11	122.81 (12)
C5—C4—H4	119.3	N2—C12—H12	118.6
C6—C5—C4	118.50 (11)	C11—C12—H12	118.6
C6—C5—H5	120.7	C10—C13—C13^i^	115.01 (13)
C4—C5—H5	120.7	C10—C13—H13A	108.5
C5—C6—C1	121.63 (11)	C13^i^—C13—H13A	108.5
C5—C6—H6	119.2	C10—C13—H13B	108.5
C1—C6—H6	119.2	C13^i^—C13—H13B	108.5
O2—C7—O1	122.52 (11)	H13A—C13—H13B	107.5
O2—C7—C1	123.52 (11)		
			
C6—C1—C2—N1	−177.85 (11)	C6—C1—C7—O1	−6.50 (16)
C7—C1—C2—N1	3.56 (18)	C2—C1—C7—O1	172.09 (10)
C6—C1—C2—C3	2.41 (17)	C12—N2—C8—C9	0.20 (18)
C7—C1—C2—C3	−176.18 (10)	N2—C8—C9—C10	−1.20 (18)
N1—C2—C3—C4	178.72 (11)	C8—C9—C10—C11	1.02 (17)
C1—C2—C3—C4	−1.53 (17)	C8—C9—C10—C13	−179.72 (11)
C2—C3—C4—C5	−0.08 (18)	C9—C10—C11—C12	0.05 (17)
C3—C4—C5—C6	0.82 (18)	C13—C10—C11—C12	−179.16 (11)
C4—C5—C6—C1	0.11 (18)	C8—N2—C12—C11	0.95 (18)
C2—C1—C6—C5	−1.75 (18)	C10—C11—C12—N2	−1.08 (19)
C7—C1—C6—C5	176.86 (11)	C11—C10—C13—C13^i^	−12.1 (2)
C6—C1—C7—O2	176.01 (11)	C9—C10—C13—C13^i^	168.71 (12)
C2—C1—C7—O2	−5.40 (18)		

Symmetry code: (i) −*x*+1, −*y*, −*z*+2.

### Hydrogen-bond geometry (Å, º)

**Table:**

*D*—H···*A*	*D*—H	H···*A*	*D*···*A*	*D*—H···*A*
N1—H1n···O2	0.86 (1)	2.03 (1)	2.6961 (15)	134 (2)
O1—H1o···N2	0.86 (1)	1.78 (1)	2.6290 (14)	172 (2)
N1—H2n···O2^ii^	0.85 (1)	2.19 (1)	3.0106 (15)	163 (1)

Symmetry code: (ii) *x*, −*y*−1/2, *z*−1/2.

## References

[bb1] Arman, H. D., Kaulgud, T., Miller, T. & Tiekink, E. R. T. (2012). *Z. Kristallogr. Cryst. Mat* **227**, 227–232.

[bb2] Arman, H. D., Miller, T. & Tiekink, E. R. T. (2012). *Z. Kristallogr. Cryst. Mat* **227**, 825–830.

[bb3] Arman, H. D. & Tiekink, E. R. T. (2013). *Acta Cryst.* E**69**, o1447. 10.1107/S160053681302271X PMC3884472 24427074

[bb4] Brandenburg, K. (2006). *DIAMOND* Crystal Impact GbR, Bonn, Germany.

[bb5] Higashi, T. (1995). *ABSCOR* Rigaku Corporation, Tokyo, Japan.

[bb6] Johnson, C. K. (1976). *ORTEPII* Report ORNL-5138. Oak Ridge National Laboratory, Tennessee, USA.

[bb7] Molecular Structure Corporation & Rigaku (2005). *CrystalClear* MSC, The Woodlands, Texas, USA, and Rigaku Corporation, Tokyo, Japan.

[bb8] Sheldrick, G. M. (2008). *Acta Cryst.* A**64**, 112–122. 10.1107/S0108767307043930 18156677

[bb9] Westrip, S. P. (2010). *J. Appl. Cryst.* **43**, 920–925.

